# The Manifestations of “l-Doubling” in Gas-Phase Rotational Dynamics

**DOI:** 10.1021/acs.jpclett.4c02918

**Published:** 2024-12-12

**Authors:** Kfir Rutman Moshe, Dina Rosenberg, Inbar Sternbach, Sharly Fleischer

**Affiliations:** § Raymond and Beverly Sackler Faculty of Exact Sciences, School of Chemistry, Tel Aviv University, Tel Aviv 6997801, Israel; ‡ Tel-Aviv University Center for Light-Matter-Interaction, Tel Aviv 6997801, Israel

## Abstract

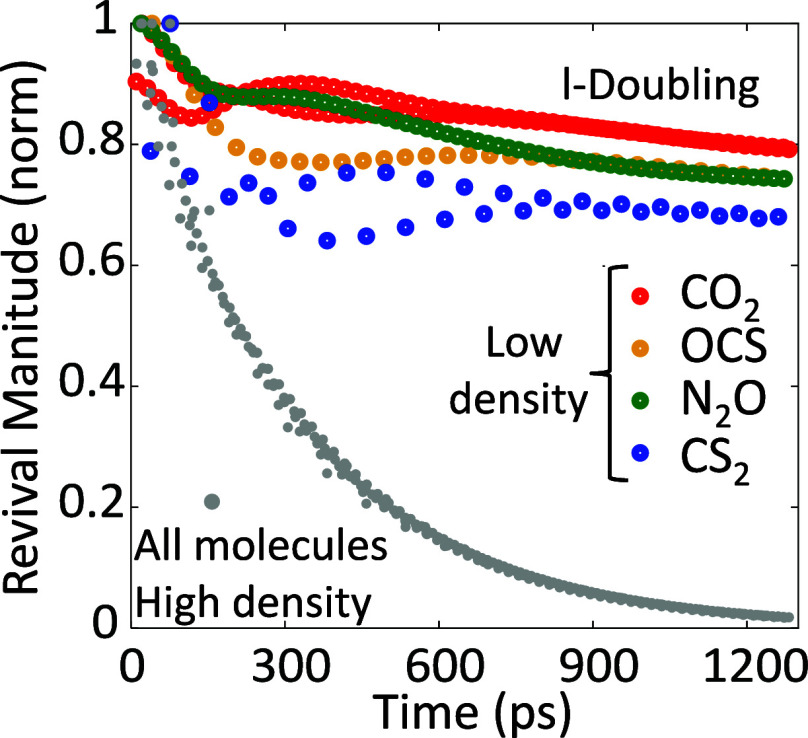

The “l-Doubling” phenomenon emanates from the coupling between molecular rotations and perpendicular vibrations (bending modes) in polyatomic molecules. This elusive phenomenon has been largely discarded in laser-induced molecular alignment. Here we explore and unveil the ramifications of l-Doubling on the coherent rotational dynamics of linear triatomic molecules at ambient temperatures and above. The observed l-Doubling dynamics may be wrongly considered as collisional decay throughout the first few hundreds of picoseconds past excitation, highlighting the importance of correct assimilation of l-Doubling in current research of dissipative rotational dynamics and in coherent rotational dynamics in general.

Upon their interaction with gas-phase molecules, ultrashort laser pulses impart short duration torque that manifests as coherent molecular rotations. This scenario has provided a rich playground for various scientific endeavors over the last 3–4 decades.^1−4^ The initial motivation of the field aimed at obtaining accurate rotational coefficients and associated molecular structure^5−9^ titled “rotational coherence spectroscopy”. The latter has evolved into coherent rotational control (“molecular alignment”), aiming to transiently lift the inherent isotropic angular distribution of gas ensembles and facilitate “molecular frame spectroscopies” via advanced optical techniques such as HHG,^10^ Coulomb explosion imaging,^11^ ultrashort X-ray diffraction^12^ and various others. In recent years, interest has gradually shifted toward the underlying processes that govern decay and decoherence phenomena in long-lasting coherent rotational dynamics. Within this realm, advanced experimental methods have been developed in the context of rotational echo spectroscopies^13,14^ and advanced modeling,^15−17^ that shed new light on the quantum mechanical aspects of rotating molecular ensembles.^18−22^

In this work, we present and explore the ramifications of “l-Doubling” on the dynamics of coherently rotating linear polyatomic molecules as revealed in molecular alignment experiments. The phenomenon was first described by Herzberg in 1942^23^ to explain the energy splitting observed in ro-vibrational spectroscopy of linear polyatomic molecules.^24^ In his paper, Herzberg coined the term “l-Doubling” in analogy to the Λ-doubling phenomenon in electronic spectra^25^ and attributed the effect primarily to the displaced position of the perpendicular vibration, which renders linear polyatomic molecules slightly asymmetric. While Herzberg mentioned that l-Doubling results, to some extent, from Coriolis coupling of different vibrations, his paper initiated a long-lasting altercation with Nielsen and Shaffer, who attributed l-Doubling solely to the Coriolis coupling.^26^ Without delving into the controversy that was beautifully detailed by James K. G. Watson,^27^ the accepted theory was given by Nielsen in 1951,^28^ who derived the expression for l-Doubling from Coriolis coupling solely.

In what follows, we provide a simplified explanation for the l-Doubling phenomenon that manifests as rotational energy splitting due to coupling with the bending modes of linear triatomics. For brevity, let us consider a molecule restricted to rotate in the xy plane. At the ground vibrational level, the rotational constant of this molecule is given by *B*_000_. Perpendicular vibration (bending) of the molecule can occur in all directions occupying the yz plane, spanned by two axial bending modes: bending along the y-axis and bending along the z-axis. Figures 1a,b depict 5 representative molecular geometries assumed by the molecule.

**Figure 1:**
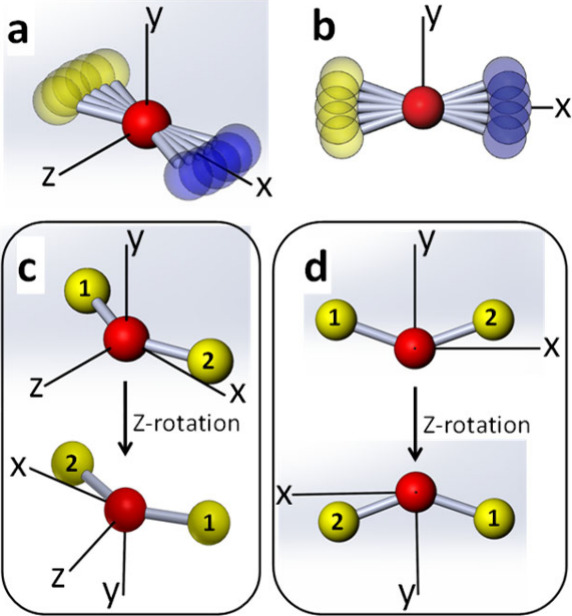
Graphical representation of molecular geometries of the two axial bending modes: (a) along the *z*-axis and (b) along the *y*-axis. Bend angles are exaggerated for better visibility. (c,d) Spatial association of the two bending modes (z-bend in fig.1c and y-bend in fig.1d) upon rotation about the *z*-axis. Relevant for linear, homonuclear triatomics —see symmetry considerations in the text related to the case of CO_2_.

Since the bending frequency (∼500 cm^–1^) is much larger than the rotational frequency (≤1 cm^–1^), we consider an effective moment of inertia for each axis by averaging over all of the geometries assumed by the molecule throughout one bending period. This results in three distinct moments of inertia:

*I*_*x*_ (which governs the rotation about the long molecular axis) is slightly shifted from its ground state value (zero) yet remains negligibly small.

*I*_*y*_ and *I*_*z*_ are both shifted to slightly lower values and their rotational constants are slightly increased. Most importantly, due to the bending of the molecule, the degeneracy of *I*_*y*_ and *I*_*z*_ is lifted and two rotational constants are formed, distinct by both their value and rotational symmetry (parity):
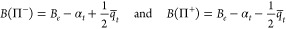
with , *B*_*e*_ the equilibrium rotational constant and ξ_*st*_ a dimensionless Coriolis coupling coefficient that is determined by the coordinates of the stretching (indexed by *s*) and bending (indexed by *t*) vibrational modes. We refer bold readers to the complete derivation by Nielsen in ref (28), where the constant α_*t*_ may be traced. In what follows we humbly proceed to the experimental manifestations of l-Doubling and corresponding simulations utilizing the experimental values of the rotational constants obtained previously (see table in Supporting Information section 1).

## Experimental Section

**Figure 2:**
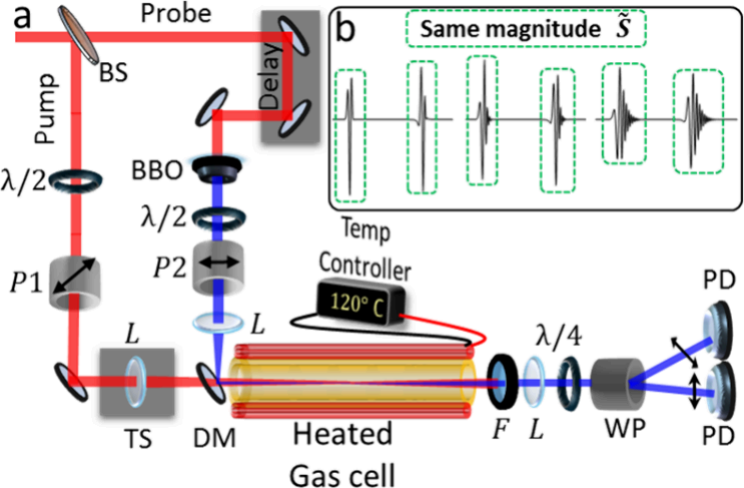
(a) Transient birefringence setup. P – polarizer, L – lens, λ/2 – half wave plate, λ/4 – quarter wave plate, BBO crystal, WP – Wollaston prism, TS – Translation stage, F – filter (BG40), PD – Photodetector. (b) Several alignment transients exemplifying the effect of centrifugal distortion. Using the quantification method (*S̃*, see text), all of these transients yield the exact same magnitude required for our analysis.

We perform time-resolved optical birefringence measurements of the anisotropic angular distributions of rotationally excited gas throughout its long field-free dynamics. Our setup (shown in Figure 2) uses the “weak field polarization detection” method^29,30^ in collinear configuration^14,18,31−33^ to extend the interaction length between the pump and probe within the gas cell to enable measurements of gas samples in the single torr level.

A femtosecond (fs) laser beam (∼110 fs, 800 nm) from a Ti:Sapphire Chirped Pulse Amplifier is split to form pump (90%) and probe (10%). The latter is frequency doubled in a BBO crystal to form a weak 400 nm probe.The timing of the probe is computer controlled using a long (50 cm, ∼1.6 ns) delay stage.

The polarization of the 400 nm probe is set at 45° to the 800 nm pump beam, and the two are combined by a dichroic mirror as in our previous work.^18^ Here, however, the pump (800 nm) and probe (400 nm) beams are focused by two selective lenses mounted on linear translation stages to enable further optimization of the overlap between the beams within the gas cell. The latter has further improved the S/N of the measurement by ∼3 fold and provided clear signals from gas densities at the level of single torr. The 800 nm pump beam is filtered out by a short-pass filter (BG40), and the transmitted 400 nm probe is analyzed for changes in its polarization by a λ/4 plate, a Wollaston prism, and a pair of balanced photodetectors.

## l-Doubling Manifestation in Rotational Decay Dynamics

**Figure 3:**
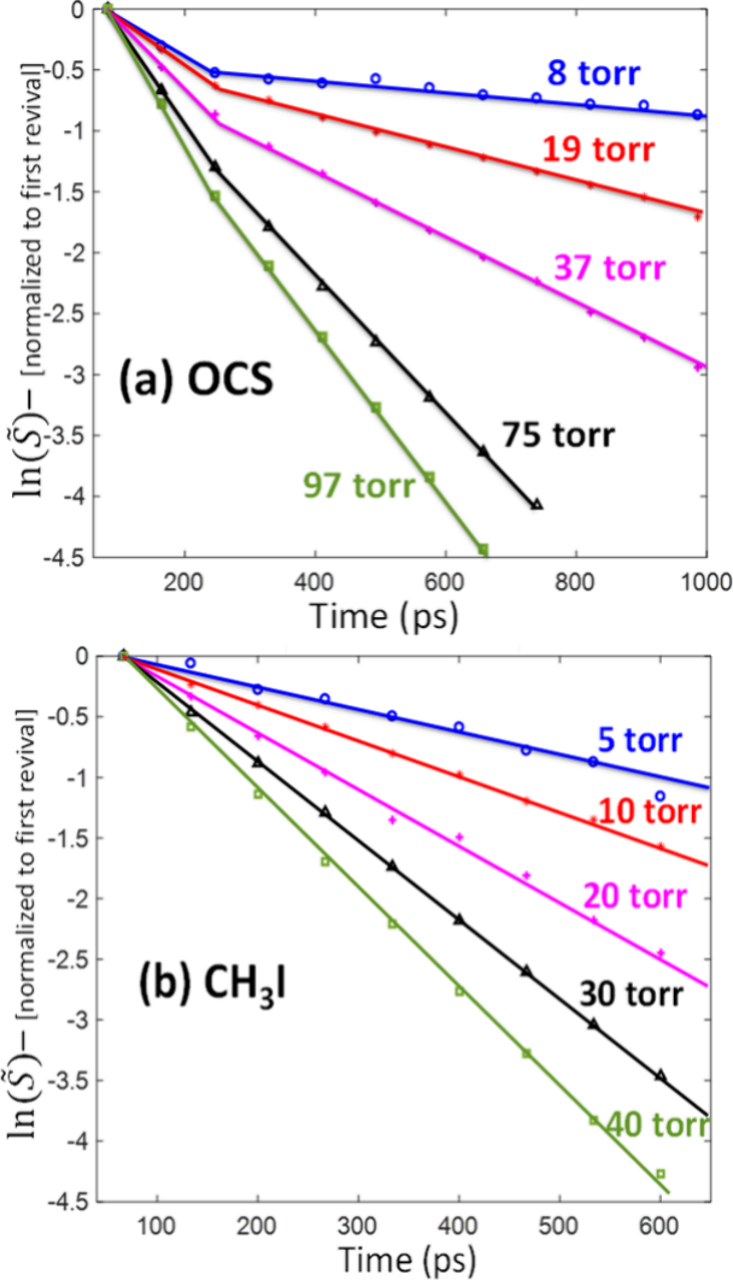
Experimental alignment magnitude (*S̃*) for (a) the OCS and (b) CH_3_I with different gas pressures at ambient temperature.

Figure 3 exemplifies the ramifications of l-Doubling by comparing the rotational decay dynamics of methyl iodide (CH_3_I, Figure 3a) and carbonyl sulfide (OCS, Figure 3b).

Each data point in Figure 3 quantifies a specific “revival signal magnitude” extracted from the time-resolved birefringence of the gas. We use a quantification metric that is immune to the centrifugal distortion dephasing used in our previous work^18,34^ and exemplified in Figure 2b where all of the alignment transients (each surrounded by dashed green line) have the exact same magnitude *S̃*, regardless of their different shapes. This is done by integrating over the power spectrum of each revival:  with ω^_1_^ = 5 cm^–1^ and ω^_2_^ = 80 cm^–1^ ( is the Fourier transform). The low frequency (ω_1_) was chosen to discard the zero frequency component that emanates from the slightly elevated birefringence background level associated with the nonthermal rotational distributions induced by the pulse. The high frequency (ω_2_) was chosen such that the range of frequencies consists most of the frequency domain of the revival transient, yet discards high frequency components that may result from measurement noise. We have tested several ranges of frequencies [ω_1_, ω_2_] with no apparent differences in the reported trends.

Each gas pressure is color coded in Figure 3 and presented on a semilogarithmic scale. In CH_3_I, the slope is purely linear (single exponential) and increases with the number density (represented by the gas pressure at ambient temperature), as expected from collision-induced decay. In OCS however, we find the decay to follow a double exponential decay, as evident by the two distinct decay slopes where the decay rate through the first three revival periods (up to ∼250 ps past excitation) significantly exceeds the collisional decay observed at longer times. While the different slopes of the two fitted linear curves are unmissable at gas pressures of 8, 19, and 37 torr, they tend to coincide as the pressure increases, and are hardly decipherable at 97 torr (green data set in Figure 3a) they are hardly decipherable. At higher gas pressures (not shown here) the dynamics can be erroneously fitted by a single exponential decay as in CH_3_I. The elusiveness of the l-Doubling phenomenon under investigation here can be appreciated by the results of Figure 3, as they are practically overshadowed by collisional decay already at subatmospheric pressures.

## l-Doubling in OCS at Different Temperatures

**Figure 4:**
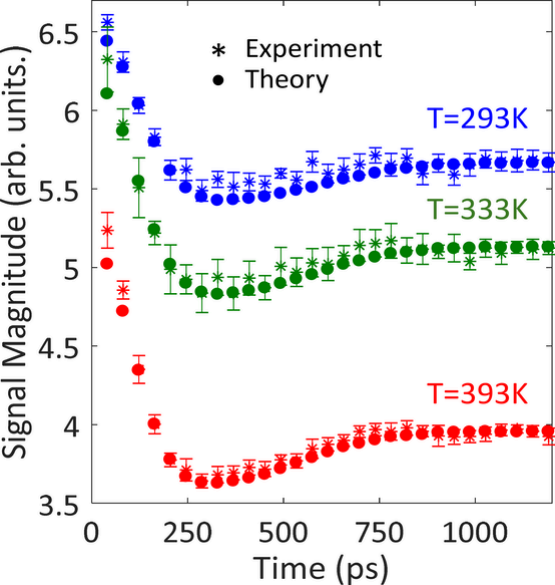
Revival signal magnitudes (***S̃***) of OCS (5 Torr gas pressure) at 293 K (blue), 333 K (green), and 393 K (red). The exponential collision-decay was extracted from the plateau region and filtered out (see text).

In order to decipher the dynamics imposed by l-Doubling from the overshadowing collisional decay, we set the experimental conditions to optimize the visibility of the former and minimize the latter. This is done by increasing the thermal population of the excited bending mode and by decreasing the gas pressure (5 torr OCS in Figure 4), respectively. Furthermore, owing to their selective temporal signatures, as shown in Figure 3a, one can decipher between the l-Doubling and collisional decay phenomena. While the first few revival periods exhibit the effects of both the l-Doubling and collisional decay, at longer delays the dynamics is governed solely by collisions. Thus, by quantifying the collisional decay rate in the region of *t* > 800 ps, we can filter out the collisional decay and effectively isolate the l-Doubling effect for the three gas temperatures depicted in Figure 4 (293 K, blue; 333 K, green; and 393 K, red). First, we calculated the magnitude of each revival transient (*S̃*) as noted above. Then we fitted the calculated magnitudes in the region *t* > 800 ps to a single exponent *S̃*_10*T*_*rev*__*e*^–*γt*^ (where *t* is in units of *T*_*rev*_ = (2*Bc*)^−1^) and extracted the collisional decay rates (γ = 6.5 × 10^–4^, 6.7 × 10^–4^ and 7.2 × 10^–4^ in units of *T*_*rev*_^–1^) for the three gas temperatures 293 K, 333 K and 393 K, respectively).

The decay-free revival magnitudes (*S̃*_*exp*_) were obtained by dividing the revival magnitudes by *e*^*–γt*^ of OCS and are marked by the color-coded asterisk symbols overlaid with the numerically simulated color-coded points.

The simulations were performed using the density matrix formalism as in our previous works.^18,35^ We numerically propagate the Liouville–Von Neumann equation, , where  is the Hamiltonian, *L̂* is the angular momentum operator, *I* is the moment of inertia and  is the interaction term for nonresonant rotational excitation with Δα the anisotropic polarizability of the molecule and |*E*(*t*)|^2^ the pulse intensity envelope. Here, however, we include the rotational contributions of molecules populating the excited vibrational states in addition to the ground state. Since the interaction term does not couple between the rotational state manifolds of different vibrational states, we can calculate the pulse-induced rotational dynamics associated with each populated vibrational level separately and sum the results weighted by their Boltzmann factor. For OCS, with ω_*bend*_ = 539 cm^–1^ we used the rotational coefficients *B*_000_ = 0.20285 cm^–1^, *B*_010_^+^ = 0.20331 cm^–1^ and *B*_010_^–^ = 0.20310*cm*^–1^ for the ground, excited in-plane and excited out-of-plane vibrations, respectively,^36^ with their corresponding thermal Boltzmann factors. The simulated alignment signal was convolved with a 350 fs Gaussian envelope to account for the length of the probe pulse measured separately in CCl_4_ reference gas (See Supporting Information section 4) and the signal magnitudes calculated (*S̃*_*sim*_). The simulated magnitudes were factored by the ratio of the experimental and simulated plateau regions (*t* > 800 ps) and resulted in very good agreement with the experimental data.

We note that we simulate the molecules as linear rotors and associate slightly different rotational coefficients with the ground and excited bending levels. This approach is found adequate at our experimental temperatures, as validated by the good agreement with the experimental results. However, it begs to reason that at highly excited bending modes (e.g., at much higher temperatures or following pre-excitation) manifested by large bending amplitudes, significant distortion of the molecular geometry may compromise the accuracy of the linear rotor model and require a more advanced treatment, e.g., the asymmetric rotor model.

Figure 4 shows that l-Doubling manifests initially as fast decay in signal magnitude at 0 → 300 ps followed by partial recovery at 300 ps → 800 ps. This is merely the beating of different revival signals with slightly different rotational coefficients associated with the three vibrational levels. Shortly after the excitation, at 1/2*T*_*rev*_ = (4*Bc*)^−1^ ∼ 41 ps, all three alignment signals are temporally synchronized; hence, they interfere constructively to yield the maximal signal magnitude. As time proceeds, they gradually desynchronize, and at ∼300 ps (∼3.5*T*_*rev*_), they interfere destructively and yield the minimal alignment magnitude. Further in time, the temporal overlap of the three contributing signals further reduces until complete temporal separation is achieved at ∼800 ps and the magnitude of the (collision-free) signal remains constant. The modulation depth, namely, the ratio between the signal dip at ∼300 ps and the first signal at ∼41 ps, depends on the relative amplitudes of the interfering signal contributions and increases with the gas temperature. At room temperature (293 K) this amounts to 16% decrease in signal magnitude, while at 333 and 393 K the signal magnitude decreases by 21% and 27%, respectively, owing to the increased thermal population of the excited bending level.

## l-Doubling in Symmetric Molecules: The Case of CO_2_

Symmetric molecules (e.g., N_2_, CO_2_, C_2_H_2_ etc.) obey the Pauli principle, which states that the total wave function of the molecule must be either symmetric or antisymmetric upon exchange of their identical Bosonic or Fermionic nuclei, respectively. For the ground electronic and vibrational states, this implies direct symmetry relations between the nuclear spin and rotational wave functions’ symmetries^37^ and enables selective rotational manipulations of ortho and para molecular spin isomers.^38,39^ Correspondingly, for CO_2_ molecules at the ground electronic and vibrational state, only the symmetric rotational states exist (with even J quantum numbers, J = 0, 2, 4, ...).^40^ The symmetry of the vibrational levels imposes additional symmetry considerations, the manifestation of which in the l-Doubling dynamics is discussed and demonstrated in what follows.

In Figures 1a,b we used a nonsymmetric triatomic model to visualize the distinct bending modes and their lifted degeneracy upon rotation, resulting in distinct *B*(Π^–^) and *B*(Π^+^) rotational coefficients. In Figures 1c,d we switch to homonuclear, symmetric triatomics (with identical nuclei, marked by “1” and “2”), where in addition to the lifted rotational degeneracy, the two bending modes are associated with different rotational symmetries. To visualize these symmetry considerations, we freeze the molecular geometry at the turning point where the molecule is maximally bent (“V” shaped) and exchange the two nuclei by rotation about the z-axis. The opposite symmetries are readily observed: while the z-bend (Figure 1c) is symmetric upon z-rotation, the y-bend (Figure 1d) is antisymmetric upon z-rotation. Correspondingly, the z-bend is associated with purely symmetric rotational levels (Even J’s) and the y-bend populates only antisymmetric rotational levels (Odd J’s). Due to their different rotational dynamics at 1/4, 3/4 *T*_*rev*_ (and recurrences),^1,38,42^ the l-Doubling manifests as partial alternations between adjacent signal magnitudes in addition to the temporally long beating dynamics.

**Figure 5:**
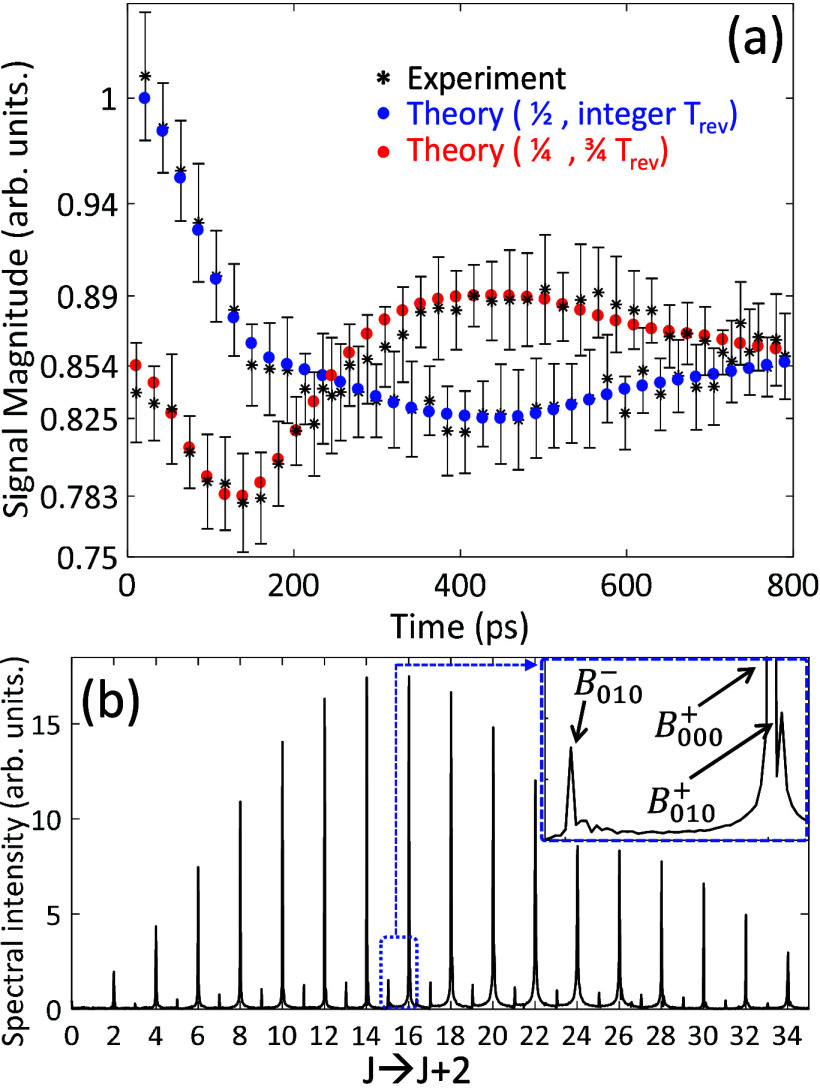
(a) Alignment magnitudes of CO_2_ gas (7 Torr, 393 K). The experimental data is given by the black asterisks overlaid with the simulated magnitudes (blue dots – half-integer revivals, red dots – quarter revivals). Note that the simulated time-dependent alignment was convolved with a 350 fs Gaussian to account for the experimental probe length (measured separately in the CCl_4_ gas sample). (b) Spectral intensity of the J → J+2 transition lines extracted from Fourier transformation of the raw time time-resolved alignment signal. The inset shows the 15 → 17 and 16 → 18 transitions where the three contributing vibrational levels are marked.

Figure 5 shows the experimental magnitudes of a CO_2_ gas sample at 393 K overlaid with the simulated results (the collisional decay was filtered out as discussed before). The half-integer revival signals  are depicted in blue and the quarter-revival signals  in red, demonstrating distinctively different dynamics. The data is normalized to the magnitude of the first blue signal  for brevity. The trend of the blue signals resembles that of OCS (Figure 4) starting with a rapid decrease in magnitude, followed by partial recovery to the level of ∼0.85 at the plateau region (*t* > 800 ps, not shown here). The red signal, however, starts at the level of the plateau (0.85) and rapidly decreases to ∼0.78 (at *t* ∼ 180 ps), after which the trend reverses and the signal increases to ∼0.89 at *t* = 440 ps (namely, above its initial level) and eventually decreases back to the plateau. These dynamics result from interference of the rotational contributions of the ground vibrational state (*B*_000_ = 0.3902 cm^–1^) with those of the excited vibrational states (*B*_010_^+^ = 0.3912 cm^–1^ and *B*_010_^–^ = 0.3905 cm^–1^).^43^ For ω_*bend*_ = 667 cm^–1^ and temperature of 393 K the Boltzmann populations are 85.2%, 7.4%, 7.4%, respectively.

In order to understand the dynamics of Figure 5, we note that the transient alignment signals coming from odd and even rotational states are π-phase shifted at quarter revivals (red signals) and identical otherwise (blue signals).^38^ In vicinity to the time of excitation, all three contributions are temporally synchronized, hence they interfere constructively at  (the first blue signal with magnitude normalized to 1). At  (first red signal), the contributions of *B*_010_^*+*^(*even J’s*) and *B*_010_^–^(*odd J*’*s*) are also synchronized, but owing to their inherent π-phase shift, they interfere destructively, leaving the *B*_000_(*even J*’*s*) contribution solely (with magnitude 0.85). As time progresses, the three signal components gradually desynchronize, owing to their different rotational constants, and the overall signal magnitude decreases. The exact time at which the blue and red signals reach their minimal and maximal magnitudes (here around *t* = 440 ps) depends on the difference in rotational constants as well on the transient signal duration. The latter is further discussed demonstrated in section 2 of the Supporting Information. We note that the minimal magnitude of the blue signal (0.825) and the maximal magnitude of the red signal (0.89) are equally distant from the plateau magnitude (0.854) due to partial temporal overlap among the three signal components that manifest in destructive and constructive interference, respectively. When the three signal components become separated in time, they do not interfere with each other any more, and the magnitude settles at the constant plateau value of 0.854.

To complete the discussion, we present the frequency domain in Figure 5b, obtained by Fourier transformation of the time-resolved alignment signal (not shown here). We plot the spectral intensity as a function of the Raman transition between the J and J+2 quantum states of the ground vibrational level using ω̃_*J*→*J*+2_ = *E*_*J*+2_ – *E*_*J*_ = *B*_000_^+^(4*J* + 6), where we discarded the centrifugal distortion for brevity. The highest populated states (high intensity peaks) are associated with the ground vibrational state (with *B*_000_^+^) and occupy even J → J+2 transitions. The odd J → J+2 transitions are attributed to the excited vibrational level (*B*_010_^–^) with low intensities (∼7.4% of the population noted above). The inset shows an enlarged view of the 15 → 17 and 16 → 18 transitions, where the latter shows the *B*_010_^+^ transition to the right of the ground state peak (marked in the inset).

In order to further verify our theoretical and experimental strategies for selectively extracting the l-Doubling dynamics presented throughout this work, we applied the procedures described above to two additional gas samples: N_2_O (nonsymmetric) and CS_2_ (symmetric). The experimental and theoretical results are provided in section 3 of the Supporting Information.

In this paper, we explored and demonstrated the ramifications of l-Doubling on the alignment dynamics of nonsymmetric (OCS) and symmetric (CO_2_) triatomic molecular models. In nonsymmetric triatomics the l-Doubling is found to (falsely) imitate a decaying dynamics at initial times past rotational excitation. In symmetric triatomics, l-Doubling dynamics is somewhat richer, with different trends observed at half integer and quarter integer revivals. The results highlight the need of l-Doubling inclusion for reliable quantification of rotational decay phenomena. The l-Doubling manifestations in rotational dynamics are easily overshadowed by collisional decay already at subatmospheric gas pressures, making l-Doubling an elusive phenomenon that may be easily overlooked. The extent to which l-Doubling affects the extracted experimental values depends on the observable of interest and on the experimental conditions. Thus, while l-Doubling may significantly alter the extracted decay rate at low gas densities, its effect may be practically negligible at high densities. As a purely coherent dephasing phenomenon, l-Doubling is coherently “rephasable” and can be deciphered from collisional decay using rotational echo spectroscopy.

## Data Availability

The data underlying this study is provided within the manuscript and Supporting Information. The raw data is available from the corresponding author upon reasonable request.

## Supporting Information Available

1) Table of rotational constants; 2) Effect of excitation pulse width; 3) l-Doubling in other gases: N_2_O and CS_2_; 4) Effect of probe duration on observed l-Doubling dynamics in OCS (PDF)
