# Supplementary material for: The Manifestations of “l-Doubling” in Gas-Phase Rotational Dynamics
Source: J Phys Chem Lett. 2024 Dec 12;15(50):12449–54. doi: 10.1021/acs.jpclett.4c02918 (PMC11664649; doi:10.1021/acs.jpclett.4c02918)
Supplement: Supplementary file 1 — jz4c02918_si_001.pdf [file jz4c02918_si_001.pdf]

Supplementary Information

The manifestations of l-Doubling in gas-phase rotational dynamics

Kfir Rutman Moshe[†1,2], Dina Rosenberg[†1,2], Inbar Sternbach, and Sharly Fleischer*[1,2]

[1]*Raymond and Beverly Sackler Faculty of Exact Sciences, School of Chemistry, Tel Aviv University 6997801, Israel.*
[2]*Tel-Aviv University center for Light-Matter-Interaction, Tel Aviv 6997801, Israel*
**Email: sharlyf@tauex.tau.ac.il*
[†]*Equal contribution*

1) Table for constants

Values are given in units of $[cm^{-1}]$

Molecule	ω_{bend}	B_{000}	B_{010}^{+}	B_{010}^{-}	q	D
CO_2	667	0.3902	0.3912	0.3905	$65 \cdot 10^{-5}$	$12 \cdot 10^{-8}$
OCS^{32}	539	0.20285	0.20331	0.2031	$21 \cdot 10^{-5}$	$4.3422 \cdot 10^{-8}$
N_2O	588.7	0.41901	0.41997	0.41917	$79 \cdot 10^{-5}$	$17.6 \cdot 10^{-8}$
CS_2	398.4	0.1091	0.10935	0.10927	$7.5 \cdot 10^{-5}$	$0.993 \cdot 10^{-8}$

Table 1: summarizing table of vibrational bending frequencies and rotational constants for CO_2[1], OCS[2] ,N_2O[3] and CS_2[4,5] molecules. Reference papers are given in brackets.

(1) Herzberg, G.; Herzberg, L. Rotation-Vibration Spectra of Diatomic and Simple Polyatomic Molecules with Long Absorbing PathsXI The Spectrum of Carbon Dioxide (Co_2) below 125μ*. *J. Opt. Soc. Am.* **1953**, *43* (11), 1037. https://doi.org/10.1364/JOSA.43.001037.

(2) Tanaka, K.; Ito, H.; Tanaka, T. Millimeter Wave Spectroscopy of OCS in Vibrationally Excited States. *J. Mol. Spectrosc.* **1984**, *107* (2), 324–332. https://doi.org/10.1016/0022-2852(84)90012-2.

(3) Plyler, E. K.; Tidwell, E. D.; Maki, A. G. Infrared Absorption Spectrum of Nitrous Oxide (N2O) from 1830 Cm-1 to 2270 Cm-1. *J. Res. Natl. Bur. Stand. Sect. A Phys. Chem.* **1964**, *68A* (1), 79. https://doi.org/10.6028/jres.068A.006.

(4) Smith, D. F.; Overend, J. General Quartic Force Field of CS2. *J. Chem. Phys.* **1971**, *54* (8), 3632–3639. https://doi.org/10.1063/1.1675389.

(5) Smith, D. F.; Overend, J. The v3 Bands of 12C32S2 and 13C32S2 at High Resolution. *Spectrochim. Acta Part A Mol. Spectrosc.* **1970**, *26* (12), 2269–2274. https://doi.org/10.1016/0584-8539(70)80178-7.

2) The effect of excitation pulse width

The l-doubling dynamics demonstrated in OCS and CO_2 naturally depends on the different rotational constants associated with the ground and excited bending modes. These rotational constants $(B_{000}, B_{010}^{+}, B_{010}^{-})$ dictate the revival periods, i.e. the times at which the three selective alignment signals will temporally overlap and interfere. The exact outcome of this three-signal interference, strongly depends on their specific shape and duration. While the shape of the alignment transient is primarily dictated by the quantum rotational dynamics, the duration of the transients can controlled by varying the duration of the excitation pulse. The interplay between the two time-scales, i.e. the (fixed) revival periodicity and the (controlled) duration of the interfering transients provides additional means to control the l-doubling temporal dynamics. In what follows, we exemplify the outcomes of this interplay by the simulations presented in figure S1. We simulate the rotational dynamics of CO_2 induced by varying excitation pulse durations. As the pulse duration increases (from 100fs to 2ps), the duration of the revival transients elongates respectively and modulation observed in the l-doubling dynamics stretches in time respectively.

This is readily observed by considering the time at which the halves and quarter revival magnitudes cross each other.

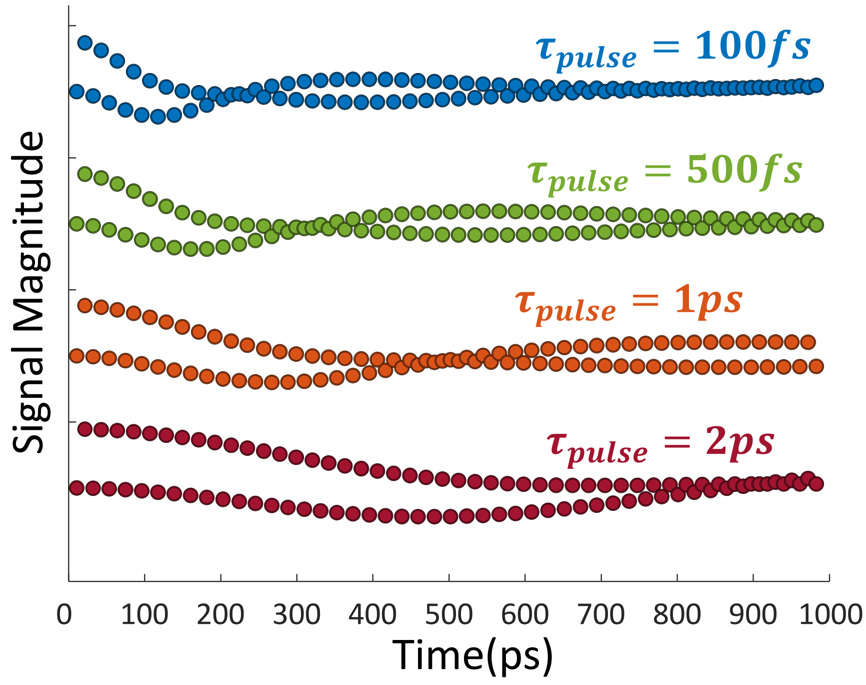

Figure S1: Simulated revival magnitudes for CO_2 for different excitation pulse durations: 0.1ps, 0.5ps, 1ps, and 2ps.

3) l-Doubling dynamics in other gas samples: N_2O and CS_2

To further validate our analysis strategy, we present the experimental overlaid with theoretical results of two additional gas species: N_2O (non-symmetric) and CS_2 (symmetric). The analysis strategy, starting from the raw optical-birefringence measurements followed by quantification of the signal magnitudes and the simulation technique is identical to that elaborated in the main paper file. Note that modulation observed in N_2O takes a different shape compared to that of OCS, and emanates from the specific values of the rotational coefficients (see table 1) and their interferences throughout the periodic revivals of N_2O.

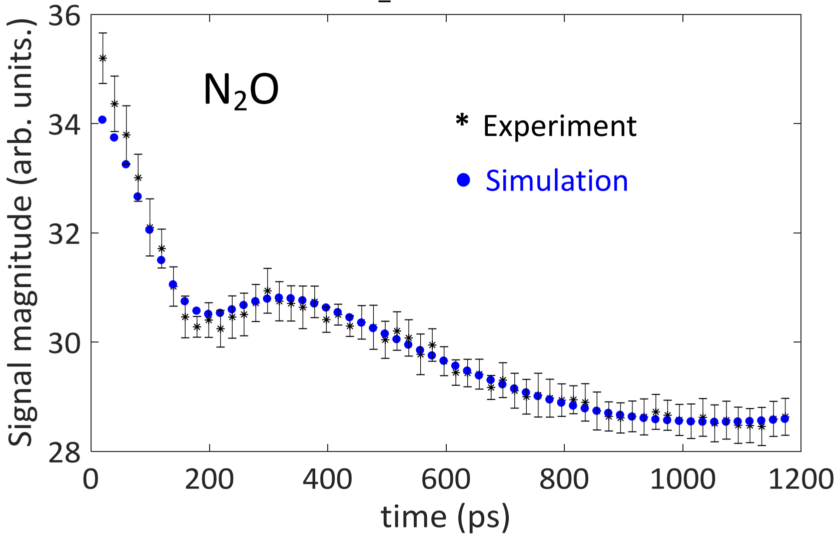

Figure S2: l-Doubling dynamics measured in 10torr N_2O sample at 333K.

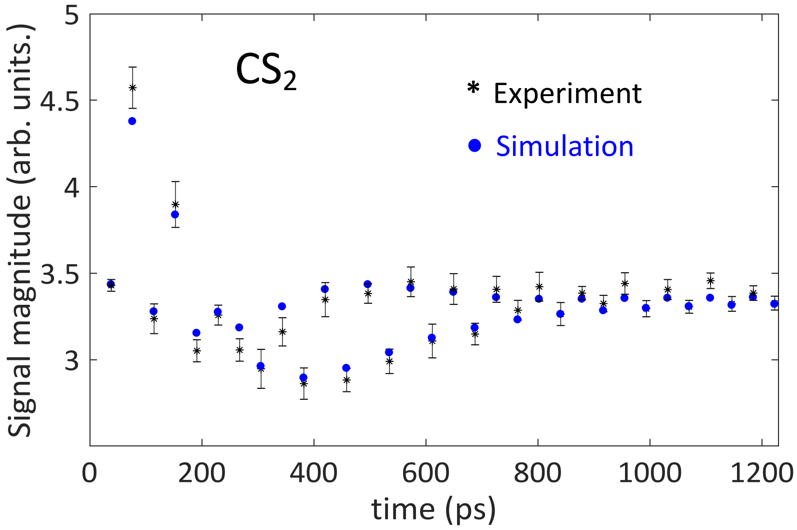

Figure S3: l-Doubling dynamics measured in 7torr CS_2 sample at 323K.

4) Effect of probe duration on observed l-Doubling dynamics in OCS

As noted in the main text, our 400nm probe duration is 350fs. This stretching is attributed to (currently unavoidable) dispersion in passing through several polarization optics and optical elements that filter-out the fundamental 800nm pulse.
While our probe duration is not optimally short, the effect of this stretching was found to be rather small as shown in Fig.S4.

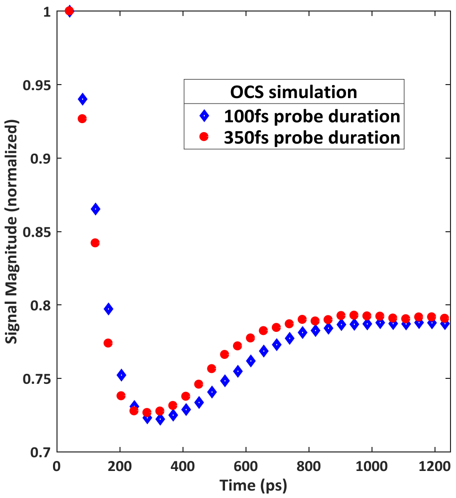

Figure S4 depicts the signal magnitudes extracted from simulated rotational dynamics of OCS. The raw data (time domain rotational revivals) were convolved with a Gaussian probe of 100fs duration and 350fs duration and the signal magnitudes calculated. To compare between the two cases we normalized the first magnitude (at t=41ps, half revival signal) obtained for 100fs probe (blue) and for 350fs probe (red). The small difference between the two is readily observed.

We note that the duration of the probe was quantified in a separate experiment using the exact same geometry and technique of our experiment. For that we filled the gas chamber with a sample of CCl_4, chosen for its isotropic polarizability to alleviate any distortions that may arise from molecular rotations. Using polarization gating provided by our experimental setup, the probe duration extracted from this scan is 350fs as stated in our paper.
